# Examining the Acceptance of Blockchain by Real Estate Buyers and Sellers

**DOI:** 10.1007/s10796-023-10411-8

**Published:** 2023-06-01

**Authors:** William Yeoh, Angela Siew Hoong Lee, Claudia Ng, Ales Popovic, Yue Han

**Affiliations:** 1 Deakin University, Geelong, Australia; 2 Sunway University, Sunway City, Malaysia; 3 NEOMA Business School, Mont-Saint-Aignan, France; 4 Le Moyne College, Syracuse, USA

**Keywords:** Blockchain, Real estate, Adoption, Factors, Partial least squares method

## Abstract

Buying and selling real estate is time consuming and labor intensive, requires many intermediaries, and incurs high fees. Blockchain technology provides the real estate industry with a reliable means of tracking transactions and increases trust between the parties involved. Despite the benefits of blockchain, its adoption in the real estate industry is still in its infancy. Therefore, we investigate the factors that influence the acceptance of blockchain technology by buyers and sellers of real estate. A research model was designed based on the combined strengths of the unified theory of technology acceptance and use model and the technology readiness index model. Data were collected from 301 real estate buyers and sellers and analyzed using the partial least squares method. The study found that real estate stakeholders should focus on psychological factors rather than technological factors when adopting blockchain. This study adds to the existing body of knowledge and provides valuable insights to real estate stakeholders on how to implement blockchain technology.

## Introduction

Real estate is very different from other assets due to high transaction costs, long-term commitment, regulations, and other constraints (Dijkstra, 2017). Buying or selling real estate is often time consuming and labor intensive, requires multiple intermediaries, and incurs high fees. High expenses include costs associated with time delays, outdated technologies, and complex data-sharing mechanisms (Latifi et al., 2019). In addition, the real estate industry faces information costs, such as the cost of coordinating trusted information between dispersed parties in relation to contract enforcement information (Sinclair et al., 2022). Blockchain technology could help the real estate industry eliminate inefficiencies and inaccuracies (Deloitte, 2019). According to transaction cost theory, adopting blockchain technology has the potential to lower real estate transaction costs and enable lower *ex-post* transaction costs by reducing verification time (Dijkstra, 2017). Combining transparent real estate markets with more effective real estate transaction processes and lower transaction costs could create more liquid real estate markets (Dijkstra, 2017).

Blockchain is a decentralized network that provides a high level of transparency and trust without the need for a central authority to vouch for accuracy (Akram et al., 2020; Kamble et al., 2019). The risk of fraud is mitigated by cryptographic signatures that make it virtually impossible to alter or forge anything registered on the blockchain (Mansfield-Devine, 2017). Blockchain can reduce effort while increasing the efficiency and effectiveness of real estate transactions. It provides the real estate industry with a reliable and transparent means to seamlessly track and trace processes (Compton & Schottenstein, 2017). Karamitsos et al. (2018) concluded that blockchain for the real estate industry could increase trust between companies involved in the real estate ecosystem and eliminates the need for intermediaries because transactions are automatically verified and validated.

Existing literature explores the benefits and applications of blockchain for the real estate industry (e.g., Konashevych, 2020; Latifi et al., 2019; Sinclair et al., 2022; Wouda & Opdenakker 2019; Yapa et al., 2018). However, despite numerous studies examining the benefits of blockchain, there is little research on how buyers and sellers perceive and accept blockchain technology in the real estate industry. Given that blockchain is an emerging technology (Akram et al., 2020), the real estate industry is still in the early stages of its adoption. More targeted studies need to be conducted on the adoption of blockchain in the real estate industry (Saari et al., 2022) because understanding blockchain adoption can help alleviate the concerns of real estate buyers and sellers, leading to broader adoption in the industry. In addition, this understanding can help real estate stakeholders and policymakers make informed decisions about how to allocate scarce resources and create relevant policies to enable blockchain implementation (Alalwan et al., 2017; Martins et al., 2014). To address this gap in the literature, we aim to investigate the factors that influence the behavioral intentions of real estate buyers and sellers in relation to the use of blockchain technology. We synergistically combine the unified theory of acceptance and use of technology (UTAUT) model and the technology readiness index (TRI) model to develop a research model and test it with real estate buyers and sellers through an online survey.

This work provides both theoretical and practical contributions. It is one of the first studies to investigate the adoption of blockchain technology in the real estate industry. It fills a gap in the literature by providing a comprehensive understanding of new technology adoption by integrating the UTAUT and TRI models. The model presented in this paper demonstrates the importance of psychological factors in technology acceptance studies and provides a new research stream for future studies. The implications for practitioners are threefold. First, a greater focus on psychological factors positively influences technology acceptance. Second, emphasizing the holistic benefits of technology in an ecosystem promotes technology acceptance. Third, forming a consortium to facilitate the technology implementation environment is beneficial when stakeholders consider new technologies.

The remainder of this paper is organized as follows. Section 2 provides an overview of blockchain for real estate and introduces the theoretical basis of this research. Section 3 provides the research model that connects the two theories and the hypotheses. The research method is then described in Sect. 4, followed by the analysis of the results in Sect. 5. Section 6 discusses the main findings of the study, the contributions of these findings to the literature, and the practical implications of the findings. Section 7 concludes the paper and suggests avenues for future research.

## Background

### Blockchain Technology and the real Estate Industry

Unlike traditional databases that are stored in a single location and controlled by a single party, blockchain is a distributed database that can store any information (e.g., records, events, or transactions) (Mougayar, 2016). Blockchain can be referred to as a metatechnology because it integrates several other technologies, such as software development, cryptographic technology, and database technology (Mougayar, 2016). Zyskind and Nathan (2015) revealed that the current practice of collecting private information by third parties poses the risk of security breaches. The main advantage of blockchain is that it can protect permanent records from data manipulation and infiltration. It also partially guarantees anonymity, transparency, transactions, and data authentication (Mougayar, 2016).

In recent years, the real estate industry has considered using blockchain technology for registering, managing, and transferring property rights (Crosby et al., 2016; Swan, 2015). Real estate industry players have recognized that blockchain-based smart contracts can help them reap the benefits of operational efficiency, automation, and transparency. Smart contracts are decentralized agreements driven by programming codes that are automatically executed when certain conditions are met (Swan, 2015). For example, if an apartment sale is handled through a smart contract, the seller gives the buyer the door code for the apartment once payment is received. The smart contract is executed and automatically releases the door code on settlement day. By using smart contracts, not only are these agreements automatically enforced, but they are also legally binding. In addition, the blockchain ensures that all actions and correspondence between buyers and sellers are recorded immutably, providing all parties with an indisputable record of payments and records (Liebkind, 2020).

According to transaction cost theory, smart contracts expedite the registration, administration, and transfer of property rights while reducing *ex-ante* and *ex-post* transaction costs (Crosby et al., 2016; Kosba et al., 2016; Swan, 2015). Smart contracts have recently become more popular because they can replace lawyers and banks involved in asset transactions according to predefined aspects (Fairfield, 2014). The use of blockchain in real estate transactions could make the transfer of money between parties faster, easier, and more efficient (Compton & Schottenstein, 2017). Blockchain application in the form of cryptocurrencies has emerged as a medium of exchange for real estate transactions, with examples in Tukwila (United States), Essex (United Kingdom), and Sabah (Malaysia) (Vanar, 2018).

Blockchain technology can transform key real estate transactions such as buying, selling, financing, leasing, and management transactions. Karamitsos et al. (2018) found that the benefits of using blockchain for real estate are that it increases trust between entities involved in real estate development and eliminates the need for intermediaries because transactions are automatically verified and validated. According to Deloitte (2019), most executives consider cost efficiency the biggest benefit of blockchain use. Table 1 provides a summary of the benefits of blockchain for the real estate industry. The table demonstrates that blockchain can reduce transaction complexity, increase security, and minimize opportunism in real estate transactions.

**Table 1:** Advantages of blockchain for the real estate industry

Advantages	Descriptions
Securing digital property records and rights system (Altynpara, 2023; Liebkind, 2020; Latifi et al., 2019; Sinclair et al., 2022; Wouda & Opdenakker 2019; Yapa et al., 2018)	• Blockchain ledger entries can record any data structure, including property titles, identity, and certification, and allow their digital transfer via smart contracts. • Blockchain can establish transparent and clear timelines for property owners. • Blockchain can automatically guarantee the legitimacy of the transfer of title. • Owners can trust that their deed is accurate and permanently recorded if property ownership is stored and verified on the blockchain because the verifiable transactional history guarantees transparency. • Blockchain serves as a single irrefutable point of truth, which can greatly benefit fraud detection and prevention, regulatory compliance, and due diligence.
Processing real estate transactions and smart contracts (Latifi et al., 2019; Sinclair et al., 2022; Wouda & Opdenakker. 2019; Yapa et al., 2018)	• Blockchain’s trustless nature allows for direct transactions between buyers and sellers, eliminating the need for external supervision of transactions. • The process can be further bolstered by implementing smart contracts that ensure a buyer–seller transaction will occur only if certain conditions are met. • Smart contracts enable the real estate to reap the benefits of deal automation and transparency. • With blockchain, trust will be in a decentralized network of actors rather than in individual actors.
Improving pre-purchase due diligence (Altynpara, 2023; Wouda & Opdenakker, 2019; Yapa et al., 2018)	• Property documents can be kept digitally in blockchain-based platforms. • These digital documents can contain all the required property data and easily be searched anytime. • The required data concerning the desired property is always accessible to every purchaser or property owner, or others involved. • Blockchain allows all paperwork to be completed automatically and can minimize the possibility of annoying paper errors and inaccuracies. • Blockchain enables realty data to be shared among a peer-to-peer network. • Blockchain enables real estate brokers to receive additional monitoring of this data and reduce their fees because data can be accessed easily.
Removing intermediaries (Yapa et al., 2018; Altynpara 2023; Latifi et al., 2019)	• Blockchain eliminates the need for intermediaries (e.g., title companies, attorneys, assessment experts, realtors/real estate agents, and escrow companies) by harnessing smart contracts. • Blockchain can become an absolute realty mediator because it can perform tasks from managing a highly secure database of property records to automatically conducting every payment.
Enabling real estate investments to become liquid through tokenization (Altynpara, 2023; Latifi et al., 2019)	• Blockchain enables real estate investments to become liquid because it provides transparent records for the desired property, secure multisignature contracts, and eliminates the need to perform tedious paperwork tasks. • Tokenization refers to the issuance of blockchain tokens acting as the digital representation of an asset or a fraction of an asset. • Tokenizing properties can bring greater liquidity to the sector, increase transparency, and make the investment in real estate more accessible.

### UTAUT

The UTAUT model suggests that four constructs—performance expectancy, effort expectancy, social influence, and facilitating conditions—are the most important determinants of intention to use information technology (Venkatesh, 2003). These constructs comprise the most influential constructs derived from eight models: the technology acceptance model (TAM); the theory of reasoned action (TRA); the motivational model (MM); the theory of planned behavior (TPB); the combined TAM + TPB (CTT); the model of personal computer utilization (MPCU); innovation diffusion theory (IDT); and social cognitive theory (SCT) (Venkatesh, 2003). Performance expectancy refers to the extent to which users expect that using the system will help them improve their job performance. This construct has four root constructs: perceived usefulness (from TAM/TAM2 and CTT); extrinsic motivation (from MM); relative advantage (from IDT); and outcome expectancy (from SCT). Effort expectancy refers to the degree of ease associated with using the system. This construct is derived from perceived ease of use (TAM/TAM2); complexity (MPCU); and ease of use (IDT). Finally, social influence indicates how significant the individual considers the use of the new system to be. This construct is represented in the UTAUT model as a “subjective norm” in TRA, TAM2, TPB, and CTT, as “social factors” in MPCU, and as an “image” in IDT. The UTAUT model is valuable in various research areas, such as continuous use of cloud services (Wang et al., 2017) and behavioral intention and use in social networking apps (Ying, 2018). In addition, the UTAUT model is more successful than the previous eight models in explaining up to 70% of use variations (Venkatesh, 2003).

### TRI

The TRI refers to the propensity of people to adopt and use new technologies to achieve their goals. The TRI can be used to gain a deeper understanding of people’s willingness to adopt and interact with technology, particularly computer and internet-based technology. Parasuraman (2000) noted that TRI can be viewed as a general state of mind that results from a gestalt of mental promoters and inhibitors that combine to determine a person’s propensity to use new technologies. The TRI has four dimensions: optimism, innovativeness, discomfort, and insecurity. Optimism is considered an indicator of a positive attitude toward technology and represents the belief that technology can bring efficiency, better control, and flexibility. Innovativeness refers to users’ inclination to pioneer technology. Discomfort describes a lack of power and a feeling of being overwhelmed when using technology. Insecurity refers to worries or distrust of the technology and its capabilities. In the four dimensions, the technology motivators are optimism and innovativeness, while the technology barriers are insecurity and discomfort. Pattansheti et al. (2016) combined TRI with TPB and TAM to explain the adoption behavior of Indian mobile banking users, and the results suggested that the integrated constructs were useful indicators. Larasati and Widyawan (2017) used TRI in conjunction with TAM to analyze enterprise resource planning implementation in small- and medium-sized enterprises and found that the combined constructs in TAM and TRI provided a better understanding of enterprise resource planning implementation.

## Research Model and Hypotheses

This study builds a research model based on UTAUT and TRI to investigate how real estate buyers and sellers perceive the use of blockchain technology. The UTAUT model presents four primary constructs that influence final intention: performance expectancy, effort expectancy, social influence, and facilitating conditions; these four constructs were included in the proposed model. Given that blockchain is still a relatively new technology that is not yet widely used in the real estate industry, the four constructs of TRI were adopted (innovativeness, optimism, discomfort, and insecurity) to explain the willingness of real estate buyers and sellers to use this technology.

Using the UTAUT model alone has the disadvantage of neglecting the psychological aspects of the user (Napitupulu et al., 2020). Previous research has demonstrated that user readiness based on personality traits is critical in driving technology acceptance (Parasuraman, 2000). The TRI is included in our study to consider characteristics that explain a person’s willingness to use technology. However, some researchers believe that TRI alone does not adequately explain why certain individuals adopt new technologies because individuals with high technology readiness do not always adopt new technologies (Basgoze, 2015; Tsikriktsis, 2004). Some previous studies have integrated the TAM model with the TRI model to combine variables on cognitive aspects and psychological traits of technology use (Adiyarta et al., 2018). However, there are few studies that examine two perspectives (technology readiness and technology acceptance) simultaneously. Examining both theories of technology readiness and acceptance simultaneously can provide a deeper description of technology adoption (Rinjany, 2020). Therefore, this study integrates the UTAUT with the TRI to complement the strengths of the two models and compensate for the weaknesses of the models. The TRI examines user readiness, while the UTAUT model examines technology acceptance factors.

**Fig. 1:**
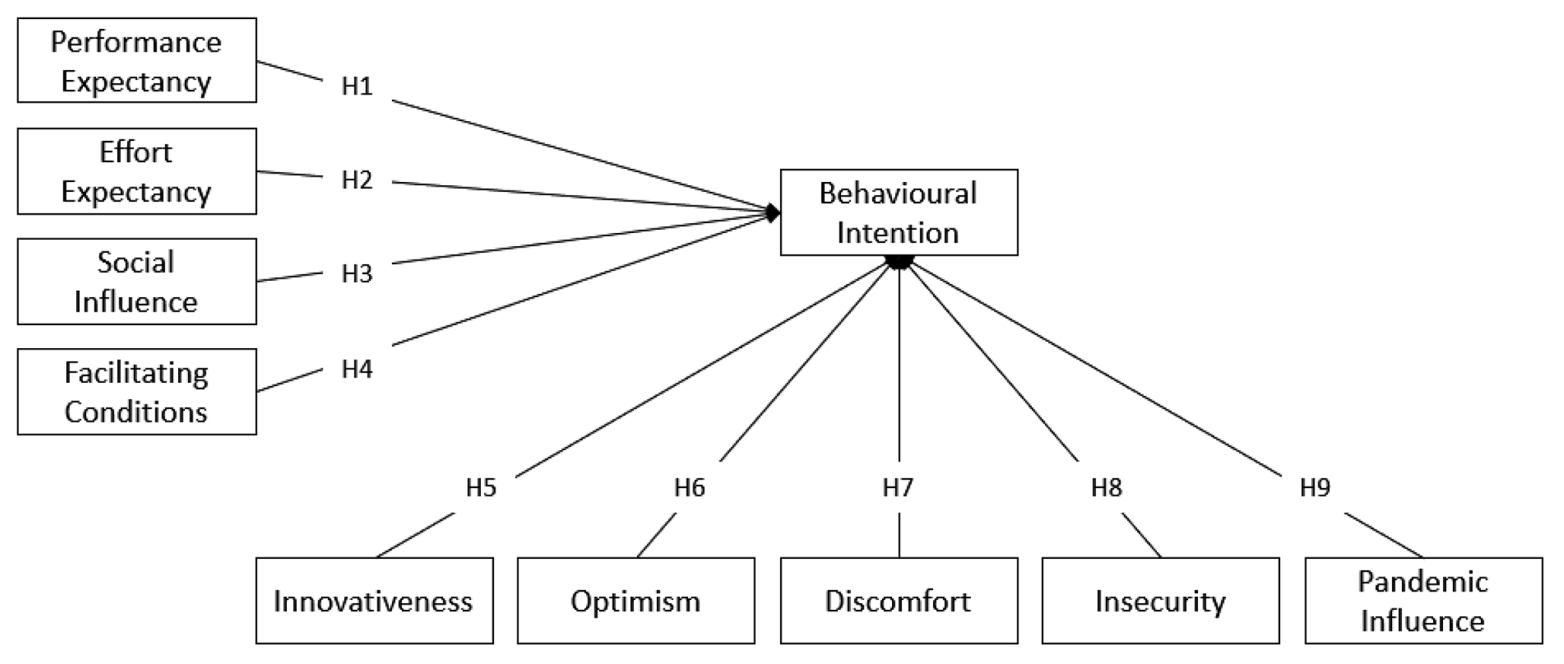
Research model

Since 2020, the COVID-19 pandemic has affected the way organizations operate and accelerated the adoption of digital technologies by several years (LaBerge et al., 2020). Because many of these changes that occurred during the pandemic (e.g., social distancing and contactless transactions) could be long term, we also include the influence of the pandemic (*PAND*) in the research model to test whether the pandemic influences respondents’ behavioral intentions to adopt blockchain. We define pandemic influence as the influence of an epidemic that occurs in a large area and affects most people. For example, physical distancing is practiced to suppress disease transmission, which leads to a contactless, paperless approach to conducting real estate transactions that do not require physical contact between real estate stakeholders becoming a priority. The research model proposed in this study is presented in Fig. 1.

### Performance Expectancy

Performance expectancy (*PEXP*) is the extent to which a person believes that the use of technology will help them improve their job performance (Venkatesh, 2003). This means that the more a user believes that a technology will improve their job performance, the greater the intention to use it (Williams et al., 2015). A person’s motivation to accept and use a new technology depends on whether they perceive certain benefits will arise from use of the technology in their daily lives (Davis, 1989). Blockchain has been shown to create high expectations for improvements in real estate transactions, such as promoting process integrity, network reliability, faster transactions, and lower costs (Latifi et al., 2019). In addition, blockchain provides liquidity in the real estate market and eliminates intermediaries through smart contracts. Previous studies have reported that the intention of individuals to accept a technology depends significantly on the expectation of performance (Alalwan et al., 2017; Riffai et al., 2012; Weerakkody et al., 2013). In this study, *PEXP* refers to the perception of a real estate buyer or seller that using blockchain would improve overall performance, including speeding up the registration and transfer of property rights, reducing the complexity of transactions with multiple parties, and eliminating the need for intermediaries in real estate transactions. Therefore, we hypothesize the following:

*H1: Performance expectancy positively affects the intention to use blockchain technology in the real estate industry.*

### Effort Expectancy

Effort expectancy (*EEXP*) refers to the ease of using a technology (Venkatesh, 2003). Individuals are less likely to use a technology if they perceive it to be difficult or if it requires more effort than to use than existing methods. Effort expectancy is closely related to performance expectancy, with the former being closer to efficiency expectancy and the latter being closer to effectiveness expectancy (Brown et al., 2010). In this study, the ease of use and complexity of blockchain can also be conveyed by the amount of time and effort required by the buyer and seller. That is, individuals will be satisfied with their experience with the technology if they perceive that it requires little effort and is low in complexity. Previous studies have demonstrated the impact of effort expectancy on the adoption of new technologies, including the blockchain (Kamble et al., 2019; Pattansheti et al., 2016). Previous research has also demonstrated that smart contracts in blockchain can minimize human effort by using predefined rules (Francisco & Swanson, 2018). In this study, *EEXP* refers to the extent to which the real estate buyer or seller feels that the blockchain is easy to use in real estate transactions. Users need to understand that the blockchain is a distributed ledger and that the smart contract is simply a program stored on the blockchain that automatically executes transactions when certain conditions are met, and they need to learn to connect the computer system to the blockchain network. Therefore, we propose the following hypothesis:

*H2: Effort expectancy positively affects the intention to use blockchain technology in the real estate industry.*

### Social Influence

Social influence (*SINF*) is the extent to which an individual perceives how significant others consider using the new system (Venkatesh, 2003). Previous research has found that social influence is exerted through the opinions of family, friends, and colleagues (Irani et al., 2009; Venkatesh & Brown, 2001). Other studies have also demonstrated that social influence factor can lead to higher intention to use when users have higher normative pressure and volume (Granovetter, 1978; Markus, 1987). The importance of social influence in accepting new technologies has also been highlighted in studies focusing on areas such as adopting mobile government services (Zamberi & Khalizani, 2017) and internet-based banking (Martins et al., 2014). In our study, *SINF* refers to how much an individual values the opinions of people around them regarding the use of blockchain in real estate transactions. Therefore, we hypothesize the following:

*H3: Social influence positively affects the intention to use blockchain technology in the real estate industry.*

### Facilitating Conditions

Facilitating conditions (*FCON*) are defined as the extent to which an individual believes that an organizational and technical infrastructure is in place to support the use of a system (Venkatesh, 2003). Facilitating conditions, such as network connectivity, hardware, and user support, have a significant impact on technology adoption and use (Queiroz & Wamba, 2019; Tran & Nguyen, 2021). Because blockchain is highly interconnected, it requires technical resources to enable its use. Insufficient resources negatively impact blockchain usage (Francisco & Swanson, 2018). For example, if there is a lack of support from the blockchain organization, users might opt for other supported systems. In contrast, if users feel that the blockchain organization provides sufficient technical support and resources, they are more likely to adopt blockchain effortlessly. From the perspective of this study, facilitating conditions emphasize the availability of the technical infrastructure and the awareness of real estate buyers and sellers about the resources available to support the use of blockchain technology in the real estate industry. Therefore, we hypothesize the following:

*H4: Facilitating conditions positively affect the intention to use blockchain technology in the real estate industry.*

### Innovativeness Users

Innovativeness (*INNO*) refers to the user’s propensity to be a pioneer in the field of technology. This factor helps to increase individuals’ willingness to accept and use technology (Parasuraman, 2000). Individuals with high levels of innovativeness are eager to try new technologies to understand new features and uses. Therefore, they are more motivated to adopt new technologies and enjoy the experience of learning them (Kuo et al., 2013). Their willingness to learn, understand, and use new technologies increases their adoption of technology (Turan et al., 2015). In addition, innovative individuals tend to be more open to new ideas and creations in general (Kwang & Rodrigues, 2002). This is also confirmed by the fact that innovativeness has been found to be a major factor influencing the intention to use technology (e.g., Buyle et al., 2018; Qasem, 2020; Zmud, 1990). In our study, *INNO* refers to the motivation and interest of real estate buyers and sellers to use blockchain for real estate transactions. Therefore, we propose the following hypothesis:

*H5: Innovativeness positively affects the intention to use blockchain technology in the real estate industry.*

### Optimism

Optimism (*OPTI*) is considered an indicator of a positive attitude toward technology. Parasuraman (2000) found that individuals who are optimistic about technology can achieve more benefits from technology in relation to control over life, flexibility, and efficiency. Scheier (1985) also found that confident and optimistic people are usually more likely to believe that good things will happen than bad things. The mindset of such people influences their attitude toward technology acceptance and risk perception (Costa-Font, 2009). These individuals have positive strategies that directly affect their technology acceptance (Walczuch et al., 2007). That is, optimistic people tend to focus less on negative things and accept technologies more readily. In this study, *OPTI* refers to the beliefs and positive attitudes of real estate buyers and sellers toward blockchain in real estate transactions. Therefore, we propose the following hypothesis.

*H6: Optimism positively affects the intention to use blockchain technology in the real estate industry.*

### Discomfort

Discomfort (*DISC*) describes feelings of lack of control and being overwhelmed when using technology. It is a barrier that lowers individuals’ willingness to use and accept technology (Parasuraman, 2000). Individuals who have high levels of discomfort with new technology are more likely to find the technology difficult to use (Walczuch et al., 2007). Discomfort indicates a low level of technological mastery, which leads to a reluctance to use the technology, ultimately making the individual uncomfortable with the technology (Rinjany, 2020). As a result, they may continue to use traditional methods to accomplish their daily tasks. Previous studies (Kuo et al., 2013; Rahman et al., 2017) have found that discomfort affects an individual’s perceived ease of use and directly influences their intention to use the technology. Given that blockchain is a new and disruptive technology, it is reasonable to assume that some discomfort will arise among individuals in relation to adopting this technology. In our research, *DISC* refers to the uneasiness of real estate buyers and sellers toward the use of blockchain in real estate transactions. Therefore, we hypothesize:

*H7: Discomfort negatively affects the intention to use blockchain technology in the real estate industry.*

### Insecurity

Insecurity (*ISEC*) refers to concern about or distrust of technology and distrust of its capabilities. Similar to discomfort, it is a barrier that lowers a person’s willingness to use and accept technology (Parasuraman, 2000). Individuals who feel less secure about technology tend to have little confidence in the security of newer technologies. Therefore, they may require more security to use new technology (Parasuraman & Colby, 2015). Distrust and pessimism about new technology and its performance can make an individual skeptical and uncertain about the performance of the technology (Rinjany, 2020). Individuals with higher levels of insecurity are more likely to be skeptical of new technologies and may not even be motivated to try them, even if they could benefit from using them (Kamble et al., 2019). Because blockchain is considered a new technology, some individuals are expected to be skeptical about it. In this study, *ISEC* refers to the distrust and uncertainty of real estate buyers and sellers about using blockchain in real estate transactions. Therefore, we hypothesize the following:

*H8: Insecurity negatively affects the intention to use blockchain technology in the real estate industry.*

### Pandemic Influence

The COVID-19 virus triggered a global pandemic that has affected all aspects of daily life and the economy. We consider the pandemic influence (*PAND*) has positively affected the use of technology in the real estate industry. According to Deloitte (2019), processes in the real estate industry are currently mainly paper based, and due diligence processes generally occur offline. Many real estate transactions (e.g., signing the letter of intent to purchase, purchase agreement, and land title registration) require face-to-face contact with stakeholders such as the buyer or seller, attorneys, and real estate agents, and require ink signatures back and forth on paper, with numerous intermediaries involved. Kalla et al. (2020) demonstrated that blockchain-based smart contracts could streamline complex application and approval processes for loans and insurance. Other benefits include eliminating processing delays caused by traditional paper-based policies and eliminating intermediaries, which typically require the physical presence of a person. As social distancing and digitization of various aspects of businesses become the norm to contain the spread of the virus (De et al., 2020), we hypothesize the following:

*H9: The impact of the pandemic positively affects the intention to use blockchain technology in the real estate industry.*

## Research Method

We developed a questionnaire based on previous literature to test the research model. The questionnaire was created using Google Forms. The participants in the survey were buyers and sellers of real estate in Malaysia. A five-point Likert scale was used, ranging from “strongly disagree” to “strongly agree”. Respondents were told they were not required to participate in the survey and that they had permission to withdraw at any time without penalty. Participants were also assured that all their data would be kept confidential. Table 2 provides the details of the measurement items.

To promote content validity, an information sheet for participants at the beginning of the questionnaire included the guidelines for the questionnaire and a request for participants to submit their responses only if they were buyers or sellers of real estate. The online questionnaire was sent to 1,000 individuals, and a total of 301 valid responses were collected, giving a response rate of 30.1%. Table 2 provides the details of the measurement items. The items were adapted from previous literature.

**Table 2:** Details of measurement items

Construct	Item	Indicator
Performance Expectancy (*PEXP*)	PE01	I would find blockchain technologies useful in real estate processes.
	PE02	Using blockchain technologies accomplishes real estate processes more quickly.
	PE03	Using blockchain technologies increases productivity in real estate processes.
	PE04	Using blockchain would improve performance in real estate processes.
	PE05	Using blockchain will help minimize transaction delays.
Effort Expectancy (*EEXP*)	EE01	I feel that blockchain would be easy to use.
	EE02	I think blockchain is clear and understandable.
	EE03	I think it will be easy for me to remember and perform tasks using blockchain.
	EE04	I feel blockchain will be easier to use compared to the conventional practices of managing real estate processes.
	EE05	I would find blockchain flexible to interact with.
Social Influence (*SINF*)	SI01	People around me believe using blockchain in real estate processes is a wise decision.
	SI02	I am more likely to use blockchain in real estate processes if people around me are using it.
	SI03	If people around me are exploring the use of blockchain, it puts pressure on me to use it.
Facilitating Conditions (*FCON*)	FC01	I know how blockchain works.
	FC03	I have the knowledge necessary to use blockchain.
Innovativeness (*INNO*)	IN01	I am open to learning new technology such as blockchain.
	IN02	I believe that it would be beneficial to replace conventional practices with blockchain.
Optimism (*OPTI*)	OP01	Blockchain would give me more control over certain aspects in the real estate processes.
	OP02	Blockchain can transform the real estate industry for the better.
	OP03	Blockchain can solve current issues faced in the real estate industry.
Discomfort (*DISC*)	DI01	It will be difficult to understand and apply the concept of blockchain in real estate.
	DI02	I think blockchain is too complex.
	DI03	There should be caution in replacing important people-tasks with blockchain technology.
	DI04	Blockchain is too complicated to be useful.
Insecurity (*ISEC*)	IS01	I consider blockchain safe to be applied in real estate.
	IS02	I am confident that sending information over blockchain is secure.
	IS03	I feel confident storing and accessing data on blockchain.
Behavioral Intention (*BINT*)	BI01	I predict that I will use blockchain in real estate processes in the future.
	BI02	I intend to use blockchain in real estate processes in the future.
	BI03	I will continuously see blockchain being used in real estate processes in the future.
	BI04	If available, I prefer blockchain to be used in real estate processes.
Pandemic Influence (*PAND*)	PAN01	I feel that blockchain could help minimize real estate sales procedures that require human contact (e.g., Smart Contracts).
	PAN02	If blockchain was implemented, it would help reduce the possible negative effects that the pandemic may have caused on the real estate economy.
	PAN03	During a pandemic, real estate sales processes would be more efficient with blockchain because it could substitute attorneys and banks involved based on predefined aspects.
	PAN04	I would feel more comfortable proceeding with selling/buying a property if blockchain was integrated in real estate processes.

## Results

Table 3 provides the demographics of the survey participants. The gender distribution among the respondents was equal, and half of the survey respondents were younger than 35 years of age. Notably, half of the respondents owned one or two properties (56.1%), followed by 17.6% who owned three or four properties, while only 4% owned five or more properties.

**Table 3:** Respondent demographics

Category	Item	Frequency	Percentage
Gender	Male	156	51.8
	Female	145	48.2
Age	< 26	76	25.2
	26–35	75	24.9
	36–45	56	18.6
	46–55	61	20.3
	> 55	33	11
Number of real estate properties owned	0	25	8.3
	0 (to purchase within the next two years)	42	14
	1 or 2	169	56.1
	3 or 4	53	17.6
	≥ 5	12	4

### Measurement Model

Measurement models indicate the relationships between constructs and the corresponding indicator variables, and the distinction between reflective and formative measures is crucial in assigning meaningful relationships in the structural model (Anderson & Gerbing, 1988). In this research, all ten constructs are reflective. The quality of the reflective measurement model is determined by the following factors: (1) internal consistency; (2) convergent validity; (3) indicator reliability; and (4) discriminant validity.

The traditional criterion for measuring internal consistency is Cronbach’s alpha (Hair et al., 2010). However, this measure is sensitive to the number of items on a scale and underestimates internal consistency reliability. Thus, it may be used as a more conservative measure. Because of the limitations of Cronbach’s alpha, it may be technically more beneficial to utilize composite reliability, which considers the different outer loadings of the indicator variables (Hair et al., 2017). Its interpretation is the same as for Cronbach’s alpha. The composite reliability of the construct should be between 0.70 and 0.95 (Grefen et al., 2000).

Given that Cronbach’s alpha is a conservative measure of reliability, and composite reliability tends to overestimate the internal consistency reliability, which could result in relatively high reliability estimates, both criteria should be considered and reported (Hair et al., 2017). Table 4 presents the Cronbach’s alpha values, composite reliability, and average variance extracted (AVE) values of all ten constructs. The Cronbach’s alpha and composite reliability values were within the threshold range of 0.70–0.95.

Convergent validity is the extent to which a measure correlates positively with alternative measures within the same construct. The common measure to establish convergent validity on the construct level is the AVE. The guideline for measuring convergent validity is that the AVE of the construct should be higher than 0.50. As presented in Table 4, the AVE value of all ten constructs meets the guideline threshold value of > 0.50.

**Table 4:** Cronbach’s alpha, composite reliability, and AVE values

Construct	Cronbach’s alpha	Composite reliability (CR)	Average variance extracted (AVE)
*BINT*	0.911	0.938	0.790
*DISC*	0.821	0.881	0.651
*EEXP*	0.919	0.939	0.756
*FCON*	0.853	0.931	0.872
*INNO*	0.729	0.878	0.783
*ISEC*	0.886	0.93	0.815
*OPTI*	0.834	0.901	0.751
*PAND*	0.845	0.895	0.682
*PEXP*	0.899	0.926	0.714
*SINF*	0.734	0.848	0.650

Note: *BINT* refers to behavioral intention

Indicator reliability represents how much variation in an item is explained by the construct and is referred to as the variance extracted from the item. To measure a construct’s indicator reliability, the following guidelines are applied: (1) the indicator’s outer loadings should be higher than 0.70 (Hair et al., 2010); and (2) indicators with outer loadings between 0.40 and 0.70 should be considered for removal only if the deletion leads to an increase in composite reliability and AVE above the suggested threshold value (Hair et al., 2017). Table 5 presents the outer loadings of all constructs. All values appear to be higher than the suggested threshold value of 0.7. Hence, no removal of constructs was required.

**Table 5:** Outer loadings

Construct	Item	Loadings
*BINT*	BI01	0.867
	BI02	0.928
	BI03	0.849
	BI04	0.909
*DISC*	DI01	0.753
	DI02	0.877
	DI03	0.715
	DI04	0.869
*EEXP*	EE01	0.886
	EE02	0.875
	EE03	0.876
	EE04	0.846
	EE05	0.866
*FCON*	FC01	0.932
	FC03	0.936
*INNO*	IN01	0.848
	IN02	0.921
*ISEC*	IS01	0.859
	IS02	0.919
	IS03	0.928
*OPTI*	OP01	0.837
	OP02	0.913
	OP03	0.848
*PAND*	PAN01	0.815
	PAN02	0.796
	PAN03	0.855
	PAN04	0.836
*PEXP*	PE01	0.877
	PE02	0.870
	PE03	0.880
	PE04	0.854
	PE05	0.737
*SINF*	SI01	0.779
	SI02	0.845
	SI03	0.793

Discriminant validity refers to how a construct is genuinely distinct from other constructs by empirical standards. To check the discriminant validity, the square roots of the AVEs were compared with the correlation for each of the constructs. The common guideline for assessing discriminant validity is that the construct’s square root AVE should be higher than the correlations between the specific construct and all the other constructs in the model (Zmud, 1990).

Table 6 presents the discriminant validity result. The diagonal items in the table signify the square roots of the AVEs—a measure of variance between the construct and its indicators—while the off-diagonal items signify the correlation between constructs. As presented in Table 6, all the square roots of the AVEs (bold) are higher than the correlation between the constructs, indicating that all the constructs in Table 6 satisfy discriminant validity and can be used to test the structural model.

**Table 6:** Discriminant validity

Construct	*BINT*	*DISC*	*EEXP*	*FCON*	*INNO*	*ISEC*	*OPTI*	*PAND*	*PEXP*	*SINF*
*BINT*	**0.889**									
*DISC*	−0.291	**0.807**								
*EEXP*	0.538	−0.346	**0.870**							
*FCON*	0.449	−0.258	0.497	**0.934**						
*INNO*	0.590	−0.142	0.387	0.330	**0.885**					
*ISEC*	−0.692	0.300	−0.466	−0.430	−0.536	**0.903**				
*OPTI*	0.673	−0.175	0.569	0.442	0.569	−0.561	**0.867**			
*PAND*	0.647	−0.156	0.465	0.281	0.558	−0.607	0.604	**0.826**		
*PEXP*	0.605	−0.208	0.584	0.356	0.543	−0.522	0.695	0.533	**0.845**	
*SINF*	0.508	−0.104	0.404	0.329	0.439	−0.446	0.485	0.457	0.582	**0.806**

### Common Method bias

Because of the self-report nature of the data collection method used in this study, common method bias may be an issue. The potential for common method bias was assessed and managed using the following measures. First, Pavlou and El Sawy (2006) asserted that common method bias results in very high correlations (i.e., r > 0.90). The highest correlation among the constructs in this study exceeded 0.90, indicating there is a concern that this study may be affected by common method bias. Thus, the Harman one-factor test was performed in which all the variables were loaded into an exploratory factor analysis. Harman’s one-factor test reveals problematic common method bias if an exploratory factor analysis returns eigenvalues that depict that the first factor accounts for more than 50% of the variance among the variables. The test result of this study indicates that the highest factor explained 27.9% of the variance among all variables, which is acceptable according to Podsakoff and Organ’s (1986) criterion. Based on Liang et al. (2007), we included a common method factor in the model. The coefficients for the measurement and structural models did not alter significantly after controlling the common method factor. Thus, we conclude that common method bias does not pose a significant threat to the results of this study.

**Fig. 2:**
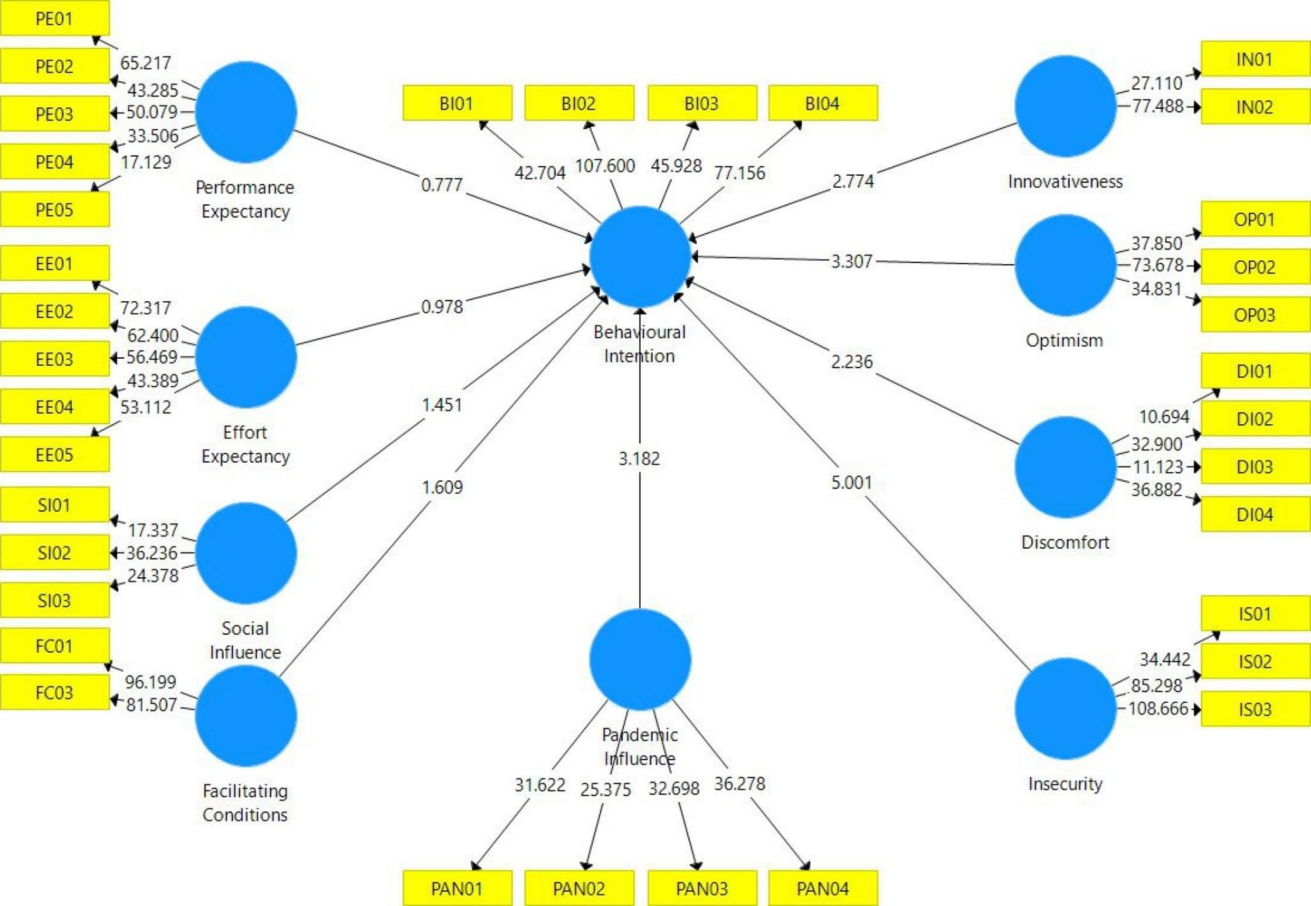
Structural model

### Structural Model

The structural model represents the underlying structural theories of the path model. The assessment of the structural model involves examining the model’s predictive capabilities and the relationships between the constructs. Figure 2 above illustrates the structural model proposed in this study. The steps for structural model assessment are as follows: (1) examine structural model for collinearity; (2) assess the significance of the path coefficients; (3) assess the level of *R*^2^; (4) assess the *f*^2^ effect size; and (5) assess the predictive relevance *Q*^2^.

The first step is to assess the collinearity between the constructs. Variance inflation factor (VIF) values of 5 or above in the construct indicate collinearity (Hair et al., 2017). Table 7 demonstrates that all VIF values of the constructs are below 5, which means there is no collinearity issue in our study.

**Table 7:** VIF values

Construct	VIF
*DISC*	1.33968
*EEXP*	2.55515
*FCON*	1.77895
*INNO*	1.57217
*ISEC*	1.69459
*OPTI*	2.45746
*PAND*	1.84538
*PEXP*	2.13851
*SINF*	1.53758

The significance of a coefficient ultimately depends on its standard error obtained through the bootstrapping procedure. Bootstrapping computes the empirical *t*-values and *p*-values for all structural path coefficients. Given that our study is exploratory, the significance level is assumed to be 10%. The bootstrapping analysis was run using a two-tailed test. Hence, the critical value is 1.65 for *t*-statistics and 0.1 for *p*-values (Hair et al., 2010). To assess the significance of the path coefficients, the guidelines are as follows: (1) *t*-value should be higher than the critical value; (2) *p*-value should be lower than 0.1 (significance level = 10%).

As presented in Table 8, *PEXP* has a nonsignificant positive effect on *BINT* (β = 0.052, *t* = 0.750, *p* = 0.454). Similarly, *EEXP* also has a nonsignificant positive effect on *BINT* (β = 0.046, *t* = 0.971, *p* = 0.332). Therefore, neither H1 nor H2 is supported.

*SINF* has a more substantial nonsignificant positive effect on *BINT* (β = 0.076, *t* = 1.460, *p* = 0.145) than the previous constructs, but it did not satisfy the minimum threshold. The same is true for *FCON*, with a stronger but nonsignificant positive effect on *BINT* (β = 0.067, *t* = 1.450, *p* = 0.148). Hence, neither H3 nor H4 are supported.

The effect of *INNO* on *BINT* (β = 0.115, *t* = 2.168, *p* = 0.009) is significantly positive. In addition, *OPTI* has a significant positive effect on *BINT* (β = 0.204, *t* = 3.431, *p* = 0.001). Therefore, both H5 and H6 are supported.

In contrast, *DISC* has a significant negative effect on *BINT* (β = −0.078, *t* = 2.251, *p* = 0.025). Likewise, the effect of *ISEC* on *BINT* is significantly negative (β = −0.273, *t* = 5.050, *p* = 0.000). Thus, H7 and H8 are both supported.

Finally, it is observed that *PAND* has a significant positive effect on *BINT* (β = 0.179, *t* = 3.389, *p* = 0.001). Hence, H9 is supported.

**Table 8:** Path coefficients

Hypothesis	Path	Path coefficient (β)		*t*-statistics	*p*-values	Hypothesis supported
H1	*PEXP -> BINT*	0.052	0.75		0.454	No
H2	*EEXP -> BINT*	0.046	0.971		0.332	No
H3	*SINF -> BINT*	0.076	1.46		0.145	No
H4	*FCON -> BINT*	0.067	1.45		0.148	No
H5	*INNO -> BINT*	0.115	2.618		0.009	Yes
H6	*OPTI -> BINT*	0.203	3.431		0.001	Yes
H7	*DISC -> BINT*	−0.078	2.251		0.025	Yes
H8	*ISEC -> BINT*	−0.273	5.05		0	Yes
H9	*PAND -> BINT*	0.179	3.389		0.001	Yes

Higher levels of the *R*^2^ value indicate higher levels of predictive accuracy. Table 9 demonstrates that the proposed model accounted for 65.7% of the variance in behavioral intention.

**Table 9:** R^2^ value for behavioral intention

Dependent construct	*R* square
*BINT*	0.657

Other than evaluating the *R*² values, changes in the *R*² value when a specified exogenous construct is excluded from the model can be used to assess whether the excluded construct has a substantial influence on the endogenous constructs. This measure is referred to as the ƒ² effect size. Guidelines for determining ƒ² are that values of 0.02, 0.15, and 0.35, respectively, represent small, medium, and large effects of the exogenous latent variable (Cohen, 1988). Effect size values of less than 0.02 indicate that there is no effect. Table 10 presents the *f*^2^ value for each variable. The values range from 0.003 to 0.105. *EEXP*, *PEXP*, *FCON*, *SINF*, and *DISC* have *f*^2^ values less than 0.02, indicating no effect. In contrast, *INNO*, *PAND*, *OPTI*, and *ISEC* have *f*^2^ values between 0.02 and 0.15, meaning these variables have a medium effect.

**Table 10:** Effect size f^2^ values

Construct	*f* ^2^
*BINT*	–
*DISC*	0.015
*EEXP*	0.003
*FCON*	0.009
*INNO*	0.021
*ISEC*	0.105
*OPTI*	0.046
*PAND*	0.045
*PEXP*	0.003
*SINF*	0.01

The predictive relevance *Q*^2^ indicates the model’s out-of-sample predictive power or predictive relevance (Geisser, 1975; Stone, 1974). A path model that exhibits predictive relevance accurately predicts data not used in the model estimation. In the structural model, *Q*² values greater than 0 suggest that the model has predictive relevance for a specific endogenous construct, whereas values of 0 and below indicate a lack of predictive relevance. As shown in Table 11, the *Q*^2^ value is 0.507, thus exceeding the minimum threshold of zero, which means that the model has predictive relevance for the construct.

**Table 11:** Predictive relevance coefficient Q^2^

Construct	*Q*²
*BINT*	0.507

## Discussions

This study combined UTAUT and TRI to develop a research model with nine hypotheses to understand the factors influencing blockchain acceptance in the real estate industry. Given that user readiness factors are explained by the TRI and technology adoption factors are explained by the UTAUT model, we integrated the UTAUT model with the TRI to complement the strengths and compensate for the weaknesses of each model. Data were collected from real estate buyers and sellers, the people most involved in and affected by buying or selling real estate. To the best of our knowledge, this study is one of the first to address the acceptance of blockchain by real estate buyers and sellers. Previous studies have examined either the technological aspect or the application of blockchain to real estate, with few studies specifically examining the adoption of blockchain in the real estate industry (Konashevych, 2020; Wouda & Opdenakker, 2019).

### Findings

This study revealed several interesting findings. The study demonstrates that four measures from the TRI model, namely innovativeness, optimism, discomfort, insecurity, and an additional measure, pandemic influence, are the most important factors affecting blockchain acceptance in the real estate industry. In contrast, four measures from the UTAUT model, namely performance expectancy, effort expectancy, social influence, and facilitating conditions, did not significantly influence the intentions of real estate buyers and sellers to use blockchain technology.

The results indicate that innovativeness positively influences the intention to use blockchain technology. This result is consistent with previous studies (Buyle et al., 2018; Qasem, 2020; Rahman et al., 2017) that have demonstrated that innovativeness has a strong influence on technology use intention. This can be explained by innovative individuals generally being more open to new ideas (Kwang & Rodrigues, 2002). Innovativeness promotes eagerness to learn, understand, and use new technologies, thus increasing technology acceptance (Turan et al., 2015). Optimism also has a positive influence on the intention to use blockchain. This finding is consistent with findings from recent studies (Koloseni & Mandari, 2017; Qasem, 2020; Rahman et al., 2017). Optimistic individuals tend to have positive perceptions of technology (Napitupulu et al., 2020). Our findings suggest that optimism increases the likelihood that individuals perceive blockchain as a technology that will improve the real estate industry.

The present study shows that discomfort hinders the intention to use blockchain technology, in contrast to some previous studies that found discomfort was insignificant in influencing blockchain adoption (Kamble et al., 2019; Pattansheti et al., 2016). However, our finding is consistent with other studies that have observed that discomfort negatively affects perceived ease of use, which directly affects technology adoption intentions (Kuo et al., 2013; Rahman et al., 2017). Given that blockchain is known as a disruptive technology, some respondents reported feeling uncomfortable that they cannot use the technology properly. Our study suggests that uncertainty affects the intention to use blockchain. This contrasts with a previous study of blockchain adoption, which found that uncertainty had an insignificant effect on perceived ease of use or usefulness on the intention to use blockchain. Most subjects did not consider the use of blockchain to be doubtful (Kamble et al., 2019). However, blockchain is seen as a new, emerging technology, particularly when considering its implementation in sectors such as real estate. As a result, uncertainty and doubt are widespread among respondents.

The results suggest that the influence of the pandemic has a positive effect on individuals’ intentions to use blockchain technology. During the COVID-19 pandemic, blockchain with smart contracts was able to simplify complicated application and approval processes for loans and insurance that were affected and extended during the lockdown periods (Pérez-Sánchez et al., 2021). That is, blockchain can mitigate the adverse effects of a pandemic situation in the real estate industry by creating smart contracts for real estate (Redolfi, 2021). Our study suggests that performance expectancy does not influence the intention to use blockchain. Furthermore, similar to previous studies, effort expectancy has no influence on intention to use, implying that effort expectancy is insignificant in determining the intention to use blockchain technology (Batara et al., 2017; Eckhardt et al., 2009). Effort expectancy and performance expectancy are closely related, with the former being more associated with efficiency expectancies and the latter more with effectiveness expectancies (Brown et al., 2010).

This study also found that social influence does not affect the intention to use blockchain, which confirms a recent study that found that social influence has no significant effect on blockchain adoption intention (Alazab et al., 2021). This result suggests that others’ experiences with blockchain acceptance do not influence real estate buyers and sellers. Moreover, we found that conducive conditions do not significantly influence behavioral intention. Previous research has found that enabling conditions influence blockchain adoption in supply chains in the United States but not in India (Queiroz & Wamba, 2019). Our study also suggests that facilitating conditions play an important role in deterring blockchain adoption in other developing countries such as Malaysia. Our research suggests that blockchain adoption by real estate buyers and sellers is mainly determined by the psychological aspects and personality traits measured by TRI rather than by the aspects of the system or technology that the UTAUT measures.

### Implications for Theory

This study provides a broader view of new technology adoption and highlights the importance of integrating the UTAUT and TRI models. Although UTAUT is a valuable model in various research areas (Venkatesh, 2003; Wang et al., 2017; Ying, 2018), the psychological aspects of the user are not considered in the model (Napitupulu et al., 2020). Our analysis demonstrates that it may be beneficial and significant to theorize about effects that are currently missing from the original UTAUT model. Integrating the constructs of the TRI model with the constructs of the UTAUT model not only enables us to examine technology readiness and acceptance simultaneously but also stimulates further research to improve existing models and deepen the study of technology adoption.

Prior studies have not attached significant importance to individual factors and major global events in influencing technology adoption and have neglected the importance of psychological factors as antecedents to intention to use information technology and systems (Adiyarta et al., 2018; Napitupulu et al., 2020). This study provides evidence that the four psychological measures of the TRI model (innovativeness, optimism, discomfort, and insecurity) all significantly affect blockchain adoption in the real estate industry. In addition, this paper shows that major global events, such as the COVID-19 pandemic, influence real estate buyers’ and sellers’ behavioral intentions to use blockchain technology. These findings provide new directions for future research, not only for the study of blockchain adoption in the real estate industry but also for the general study of technology adoption.

### Implications for Practice

This paper also has important implications for practitioners. The first implication is that it would be beneficial for blockchain and real estate stakeholders to focus more on psychological factors than technological factors when implementing blockchain. They can conduct pre-implementation studies, such as surveys or focus groups, to understand personal characteristics and address potential psychological concerns, which will help improve the efficiency of technology adoption when implementing revolutionary blockchain technology.

The second implication for real estate stakeholders is that emphasizing the holistic benefits of blockchain technology to the real estate ecosystem, including buyers and sellers, is more likely to drive technology adoption than outlining blockchain’s features. As our study shows, people are more experienced in using various new technologies in today’s internet age. Therefore, performance expectancy and effort expectancy were not found to be critical in influencing users’ intentions to use blockchain. In contrast, knowledge of the holistic benefits may contribute to psychological factors that positively impact technology adoption, such as innovativeness and optimism, and mitigate the negative psychological factors, such as discomfort and insecurity.

The third implication is that stakeholders in the real estate industry, such as professional associations, government agencies, financial institutions, brokers, and lawyers, should collaborate to establish a blockchain network so that real estate settlements can be conducted online with smart contracts and blockchain-based streamlined processes. The three implications of this study can also provide stakeholders in sectors other than real estate with insights into adopting new technologies.

### Limitations and Future Research

Like any other study, this study has limitations that provide further research opportunities. First, our model was tested in Malaysia, which is a developing country. Future studies can apply a comparative research approach and test our model in developed countries. Second, our study is limited to the real estate industry. Researchers can further investigate the acceptance of blockchain technology by applying our research model to other sectors or industries.

## Conclusion

Based on the UTAUT and TRI models, this paper conceptualized and empirically examined the factors that influence intentions to use blockchain technology in the real estate industry. Data were collected from 301 real estate buyers and sellers and analyzed using the partial least squares method. The results showed high internal consistency and reliability, indicating that the study has high predictive accuracy. The study concluded that the intention of real estate actors to use blockchain is significantly influenced by the following factors: innovativeness, optimism, discomfort, insecurity, and pandemic influence. Thus, our empirical investigation shows that the model we propose, which reformulates the theses of the original UTAUT model, can provide a useful alternative for understanding blockchain acceptance and use.
